# Assessing Video Presentations as Environmental Enrichment for Laboratory Birds

**DOI:** 10.1371/journal.pone.0096949

**Published:** 2014-05-14

**Authors:** Marion Coulon, Laurence Henry, Audrey Perret, Hugo Cousillas, Martine Hausberger, Isabelle George

**Affiliations:** UMR 6552 – Ethologie Animale et Humaine, Université Rennes 1 – CNRS, Rennes, France; Université Pierre et Marie Curie, France

## Abstract

The aim of the present study was to investigate the effects of video presentations of natural landscapes on European starlings' (*Sturnus vulgaris*) stereotypic behaviours (SBs) and other abnormal repetitive behaviours (ARBs) and to evaluate the impact of past experience by comparing wild-caught and hand-reared starlings' reactions. Ten wild-caught and five hand-reared starlings were presented 1-hour videos of landscapes twice a day for five successive days, while a control group of eight wild-caught and four hand-reared starlings was presented a grey screen for the same amount of time. The analysis of the starlings' behaviour revealed that the video presentations of landscapes appeared to have a positive but limited and experience-dependent effect on starlings' SBs and other ARBs compared to the controls. Indeed, whereas video presentations seemed to modulate high rates of SBs and ARBs, they did not appear to be enriching enough to prevent the emergence or the development of SBs and ARBs in an impoverished environment. They even appeared to promote a particular type of SB (somersaulting) that is thought to be linked to escape motivation. The fact that this effect was observed in hand-reared starlings suggests that videos of landscapes could elicit motivation to escape even in birds that never experienced outdoor life. These results highlight the importance of investigating stereotypic behaviour both quantitatively and qualitatively in order to provide crucial clues on animal welfare.

## Introduction

Captivity generally offers restricted living conditions that can, over time, lead to welfare problems [1], [2]. For several decades, strategies such as restoring appropriate environmental, social or feeding conditions have been developed to deal with these welfare problems (e.g. [3]–[5]). Recently, the use of human-created artificial stimuli, such as music (e.g. [6], [7]) or video/television (e.g. [8]–[10]), has been found to be relatively efficient environmental enrichments for some mammal species (for a review, see [11]). Both chicks and hens appear to be attracted to video images [12], [13], and regular exposure of chicks to video stimulations can reduce their fear of a novel environment [14]. One can therefore wonder whether video stimulations may be used as environmental enrichment for laboratory birds.

One particular way of expressing impaired welfare is stereotypic behaviour (SB), which is absent under natural conditions but expressed by a variety of species when placed in restricted conditions such as farms, zoos or laboratories [4], [15]. Although usually defined as repetitive, invariant behaviour patterns that have no obvious goal or function (e.g. [16]), stereotypies are diverse and heterogeneous and there is no clear-cut distinction between what is truly stereotypic and what is not [15]. However, SBs and other abnormal repetitive behaviours (ARBs) are generally considered to be associated with poor welfare and are suggested to be a way for animals to cope with unfavourable stress-inducing environments [1], [2]. Linked with feeding, social or spatial frustration (e.g. [17], [18]), laboratory birds frequently express SBs and ARBs [16], [19]–[21]. In European starlings, *Sturnus vulgaris*, the most common SB is somersaulting. This behaviour is thought to develop from thwarted escape attempts that become chronic [19], [20], [22]. Interestingly, SBs and other ARBs differ according to starlings' previous experience: wild-caught starlings are more likely to develop ARBs than hand-reared starlings are [23], [24].

The present study aimed at evaluating the effects of video presentations of natural landscapes on captive starlings' behaviour. European starlings are widely used in laboratory research, and they are most of the time housed singly or in small groups [25]. Their welfare should therefore be of prime concern for a number of scientists. Moreover, as starlings are songbirds that are widely studied for their song behaviour, it is common for them to be placed in soundproof chambers that allow song recordings. Since soundproof chambers are secluded and confined environments that are not likely to promote welfare, looking for possible enrichment in this context is important. However, to date, no study clearly investigated whether videos could be a good enrichment for this species. We thus examined the effects of video presentations of natural landscapes on starlings' behaviour. We expected the videos to create an illusion of outside window or a diversion that would decrease the starlings' frustration, reduce their motivation to escape, and consequently the associated SBs and other ARBs. We also evaluated the impact of starlings' previous experience on their reactions to video stimulations by testing not only wild-caught starlings but also hand-reared starlings. According to Feenders and colleagues [23], [24], hand-reared starlings respond less to various stressors, in particular to their introduction into a novel environment, than wild-caught starlings do. They would also be less prone to develop somersaulting because of lower escape motivation in a small confined space. We thus predicted that our hand-reared starlings would perform less SBs and other ARBs than the wild-caught starlings at the beginning of the experiment, when exposed to a novel environment. However, since hand-reared starlings had never been outdoors, the illusion of outside window might not be effective. We therefore did not expect any long-term effects of the videos on their behaviour.

## Methods

### 1) Ethics statement

This study was performed in Rennes, France (license number 35-238-15 and license number 35–119 issued by the departmental direction of veterinary services of Ille-et-Vilaine), in accordance with the European Communities Council Directive of 24 November 1986 (86/609/EEC). It was approved by the local Ethic Committee for Animal Experimentation (“Comité Rennais d'Ethique en matière d'Expérimentation Animale”, registered with the National Ethic Committee for Animal Experimentation as number 07).

### 2) Subjects and housing conditions

Eighteen wild-caught and nine hand-reared male European starlings were used in this study. All of them were at least 1 year old at the time of the experiment. The wild starlings were caught with mist nets at the time of their autumnal migration in 2006 (Normandy, France). Immediately after the capture, they were ringed with a unique combination of coloured rings for identification, and transferred to a large outdoor aviary (18×7.5×2.5 m) equipped with perches. They were kept in this aviary as a mixed-sex group during 4–5 years, until the experiment took place in 2010/2011. All birds were provided with food (commercial pellets) and water ad libitum.

The hand-reared starlings were taken from wild nests in May 2009, 1 week post-hatching. They came from different broods of sedentary colonies in Rennes (France). They were transferred to the laboratory for hand-rearing, and housed in artificial nests lined with tissue paper. The chicks were initially fed every 30 minutes during 14 hours per day. The frequency of feeds was gradually reduced as the birds grew. When about 1 month old, when the chicks fledged and started to feed themselves, they were ringed for individual identification and transferred to an indoor aviary (2.1×1.05×2 m) equipped with perches. Food (commercial pellets) and water were provided ad libitum. Lighting was adjusted weekly in order to follow the natural local photoperiod.

### 3) Experimental design

Each of the 27 males was housed singly in an individual cage (60×39×65 cm) placed in a solid-sided sound-proof chamber (sound attenuation of 35dB). Subjects could therefore neither see nor hear each other, which precluded interactions between individuals. Since the aim of the study was to evaluate the enriching power of a device, placing the subjects in an impoverished environment was required. Hand-reared starlings had never been housed in soundproof chambers before the experiment, whereas 11 of the 18 wild-caught starlings had already experienced temporary housing in soundproof chambers. No difference was observed between wild-caught starlings who experienced temporary housing in soundproof chambers and those who didn't. Each cage contained two perches, two food dispensers, and two water dispensers. Food (commercial pellets) and water were provided ad libitum. Lighting was adjusted weekly in order to follow the natural local photoperiod.

Each cage was equipped with a 15''LCD colour monitor (NEC AccuSync LCD52VM). This screen, fixed on a wall inside the cage and protected by a transparent acrylic glass (37.5×62.5 cm), was connected to a computer for the video presentation. A camera (Kodak Zi6), placed in a corner of the sound-proof chamber (outside the cage), recorded the birds' behaviour during the experiment.

### 4) Stimuli

**Figure 1:**
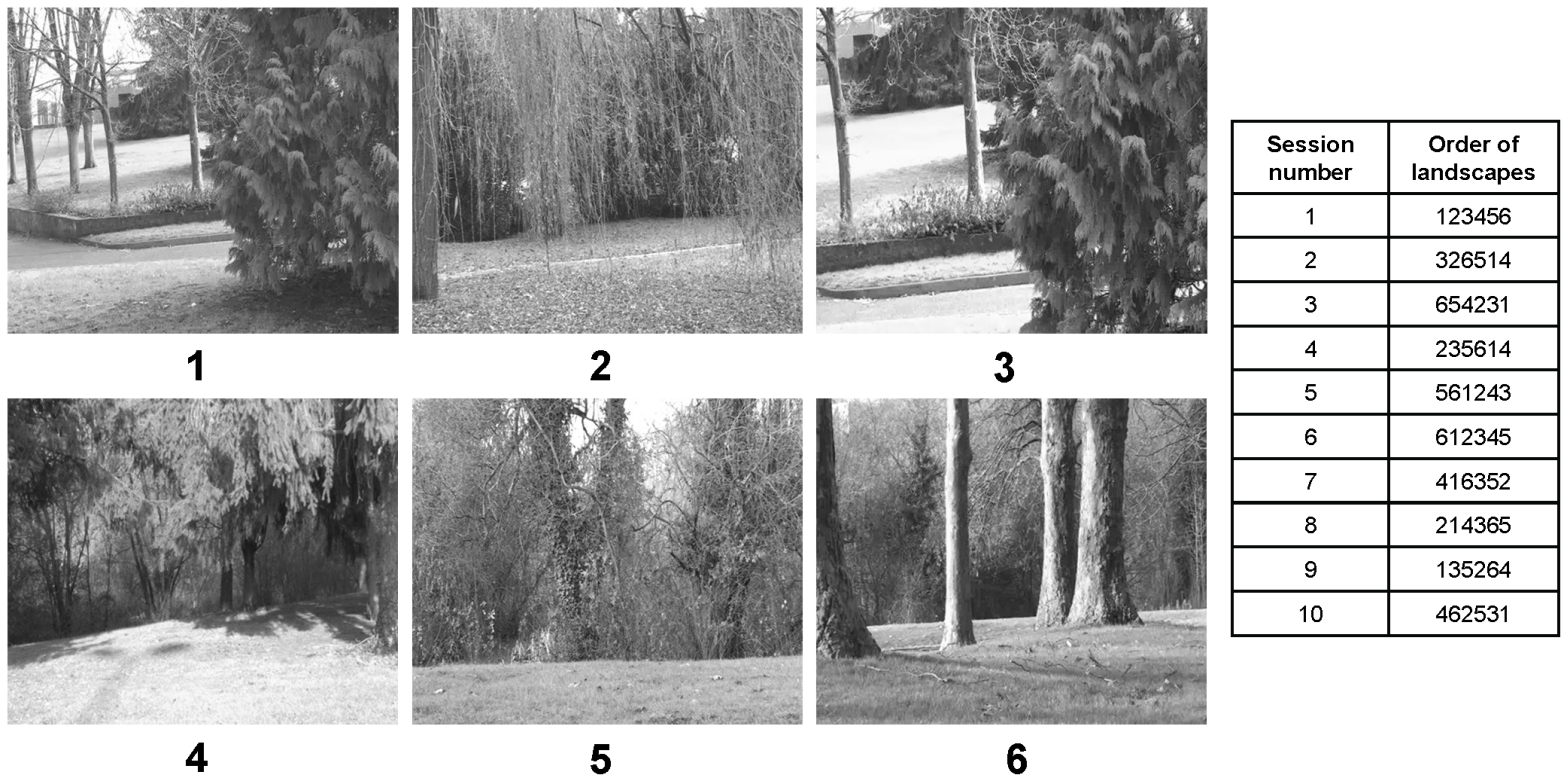
Screenshots of the six landscape sequences used to create the one-hour video films, and order of presentation of the different landscapes throughout the experiment. Although the pictures here are in black and white, they were projected in colour during the experiments.

Ten 1-hour colour video films, each presenting a succession of landscape sequences, were specially created for this study. For that, six 10-minute sequences of different natural landscapes (Figure 1) were recorded on the campus of Rennes 1 University (France). They were then assembled to create 1-hour films. Each of the ten films presented the landscape sequences in a different order (Figure 1), so that the birds saw a different presentation at each session of the experiment. All the video films were deliberately mute. We chose sequences with slight motion (e.g. branches lightly swaying in the wind) so that the starlings did not perceive the landscapes as completely static. We ensured that the videos contained neither animals, nor humans, nor vehicles (cars, bicycles…). Although we do not know how starlings actually perceive videos, there is a large amount of evidence that suggests that a variety of avian species show natural behaviour and even individual recognition when confronted with videos (e.g. rooks: [26]; quail: [27]; zebra finches: [28], [29]). Moreover, although pictures are not the same as video images, the fact that starlings are able to discriminate pictures of landscapes from pictures of conspecifics (unpublished data) and pictures of familiar conspecifics from pictures of unfamiliar conspecifics [30] shows that TFT screens are not a problem for them.

### 5) Protocol

After a day of habituation in the soundproof chamber, the experiment began. Ten wild-caught and five hand-reared starlings (experimental group) were exposed to the 1-hour landscapes videos twice a day, from 10:00 am to 11:00 am and from 3:00 pm to 4:00 pm (GMT+1), for five successive days, while four wild-caught and four hand-reared starlings (control group) were exposed to an unchanging blank screen of grey, for the same amount of time and with the same time schedule as the experimental group. Each subject's behaviour was recorded by a camera during these presentations.

Although five days may appear as a rather short period of time, it has been shown that starlings can exhibit SBs within a week of being placed in a cage [20]. Moreover, this time span was sufficient for us to observe significant effects of the video presentations compared to the controls and significant changes between the first and the last days of the experiment (see results).

### 6) Behavioural measurements

We focused our analysis on the first and the last day of the experiment (i.e., the first two and the last two sessions of video presentations). 92 hours of video recordings were thus analysed (i.e., 4 hours per bird). Instantaneous scan sampling of each starling's activity was performed every 10 seconds as in previous studies (e.g. [31]). The sampling started at the beginning of the video recording and starlings' behaviour was subsequently noted every ten seconds. Using instantaneous scan sampling of each starling's activity every 10 seconds to analyse a whole 1-hour session gave the same results as sampling all occurrences during 10 minutes of this session. We therefore chose to use the method that allowed us to scan the whole duration of each session. We identified six types of abnormal repetitive behaviours. One of these behaviours was a sequence that is well-known in captive starlings and was therefore considered as a stereotypic behaviour (SB). Other sequences that were less or not described or recognized were simply considered as abnormal repetitive behaviours (ARBs). Most of these behaviours have been already described by other authors [19], [20], [22]. We describe below the only SB and the 5 ARBs that we observed.

SB:

- Somersault: the starling left the floor/perch and turned forwards or backwards in a complete revolution in the air bringing its feet over its head, unless it held on the ceiling during the movement. Loops and falls as defined by Feenders and Batesons [20] were considered here as somersaults. Somersaulting is a commonly reported stereotypy in starlings [20].

ARBs:

- Repetitive cage perching: the starling clung repeatedly on to a side of the mesh rectangular cage with its claws. Hanging on the cage is considered as indicative of escape behaviour [22].

- Head tilting: the starling tilted its head back such that its bill broke the vertical plane. Head tilting is considered as a precursor of somersaults [20].

- Repetitive screen pouncing: the starling threw itself repeatedly on the screen by flying or jumping against it.

- Repetitive pecking: the starling pecked repeatedly at the cage with its beak closed. Pecking at the cage is considered as indicative of escape behaviour [22].

- Wing tremble: the starling suddenly shook its wings with quick, short movements.

### 7) Data analysis

Non-parametric statistical analyses were used with an accepted *p* level at 0.05. Chi-square (*χ^2^*) tests and Wilcoxon signed rank tests were used to compare SBs and ARBs rates between the first and the last day of the experiment for each individual and group, and Mann-Whitney U-tests were used to compare wild-caught and hand-reared starlings. Kendall rank correlation coefficients were used to compare the relative rates of the different types of SBs and ARBs at the beginning and at the end of the experiment, independently for each group.

## Results

### 1) Quantitative analysis

#### (i) Wild-caught starlings

**Figure 2:**
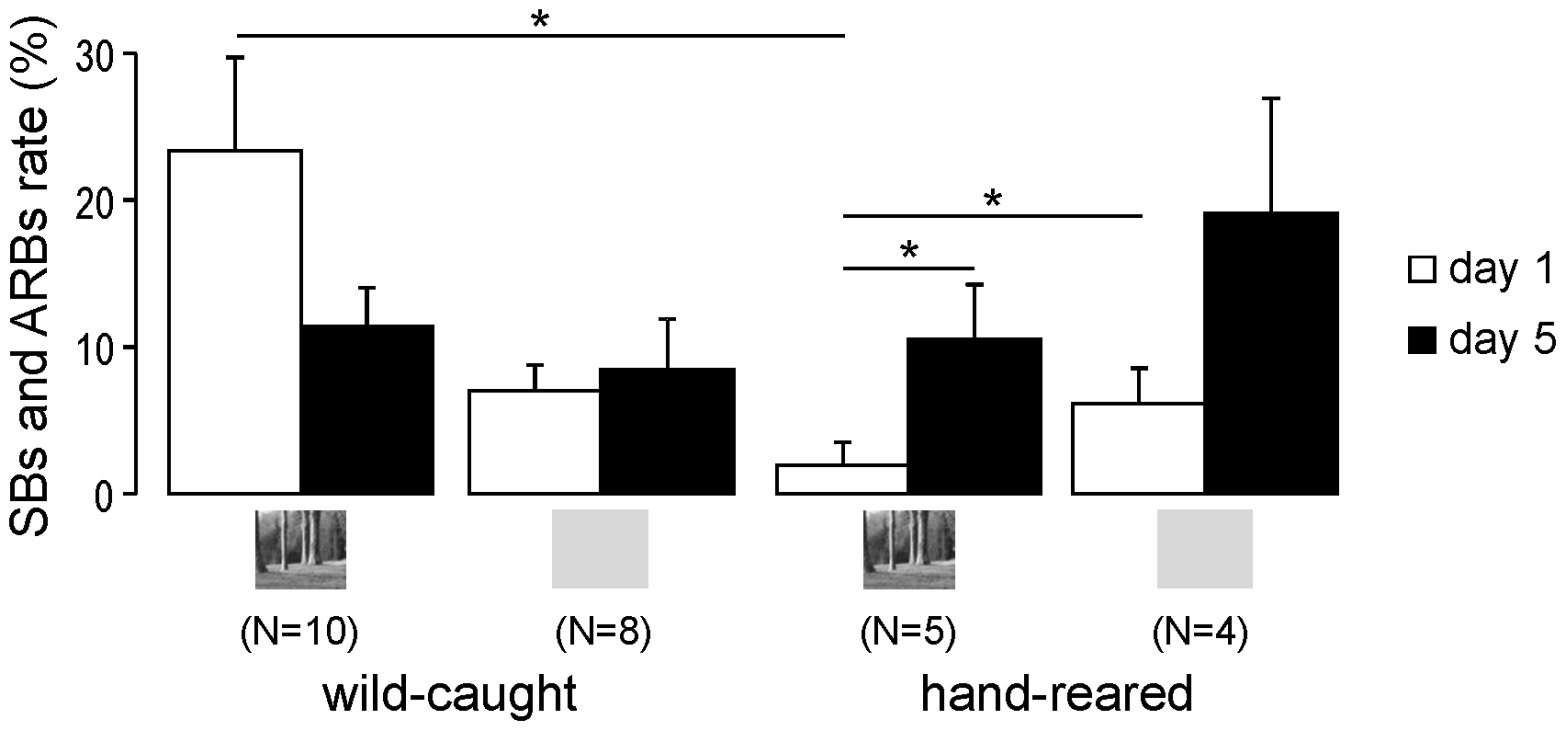
Stereotypic behaviours (SBs) and other abnormal repetitive behaviours (ARBs) rate (mean percentage + SE) on the first (white bars) and on the last day (black bars) of the experiment for experimental and control wild-caught and hand-reared starlings. Small inserts below the x axis indicate whether the birds were presented landscape videos or grey screen.

Wild-caught starlings' mean rates of SBs and ARBs did not differ significantly between the experimental and the control groups on the first day (respectively, *M*±*SE* = 23.34±6.40% and 6.98±1.78%, Mann-Whitney U test, *p* = 0.07) or on the last day of the experiment (respectively, *M*±*SE* = 11.4±12.61% and 8.45±3.46%, Mann-Whitney U test, *p* = 0.42), and stereotypy rates of both groups did not change significantly between the first day and the last day of the experiment (Wilcoxon signed rank test, *p* = 0.10 for the experimental group and 0.50 for the control group) (Figure 2).

**Figure 3:**
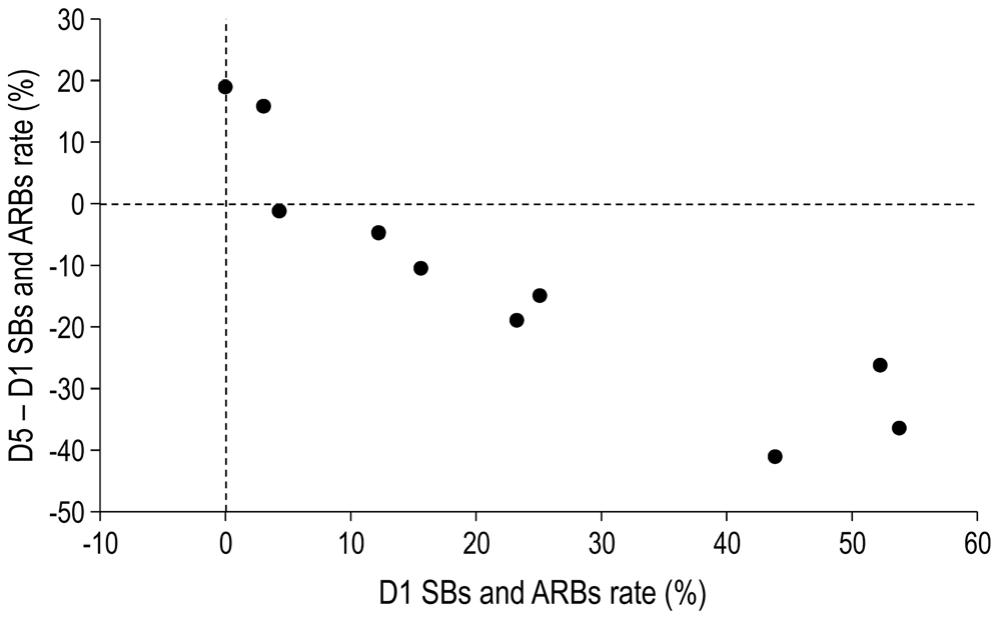
Correlation between the SBs and ARBs rate on the first day (D1) of the experiment and the difference in this rate between the last day (D5) and the first day of the experiment in the experimental wild-caught starlings. Each dot corresponds to one starling.

Although all subjects but two exhibited SBs and ARBs, inter-individual variations were important in both groups (Table 1). Analysis of individual values revealed that SBs and ARBs rates decreased significantly between the first day and the last day of the experiment for 7 of the 10 starlings of the experimental group (Table 1). By contrast, only 2 of the 8 starlings of the control group showed a significant decrease in their SBs and ARBs rates between the first day and the last day of the experiment (Table 1). In the experimental group, the higher the SBs and ARBs rates were on the first day of the experiment, the higher the decrease between the first day and the last day of the experiment was (Spearman correlation, rho = −0.952, p = 0.004; Figure 3).

**Table 1:** Variations of individual SBs and ARBs rates (%) between the first day (day1) and the last day (day 5) of the experiment for experimental and control wild-caught and hand-reared starlings.

			day 1	day 5	Δ	*χ^2^*	*p*	
Wild-caught starlings	exp.	**A**	**0.0**	**19.0**	**19.0**	**136.50**	***<0.001***	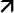
		**C**	**3.1**	**18.9**	**15.8**	**82.25**	***<0.001***	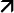
		D	4.3	3.1	−1.2	1.48	*>0,05*	=
		**E**	**12.2**	**7.5**	**−4.7**	**8.14**	***<0,01***	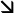
		**B**	**15.6**	**5.1**	**−10.5**	**37.81**	***<0.001***	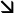
		**N**	**23.3**	**4.3**	**−19.0**	**89.39**	***<0.001***	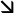
		**M**	**25.1**	**10.1**	**−14.9**	**32.70**	***<0.001***	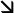
		**L**	**43.9**	**2.8**	**−41.1**	**257.84**	***<0.001***	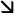
		**J**	**52.3**	**26.0**	**−26.3**	**59.58**	***<0.001***	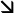
		**K**	**53.8**	**17.4**	**−36.4**	**102.56**	***<0.001***	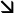
	control	F	0.0	0.0	0.0	/	/	=
		Q	0.0	0.0	0.0	/	/	=
		**P**	**4.3**	**10.7**	**6.4**	**19.59**	***<0.001***	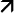
		I	7.8	7.8	0.0	0.00	*>0,05*	=
		**H**	**9.4**	**21.3**	**11.8**	**30.02**	***<0.001***	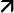
		**R**	**9.4**	**3.3**	**−6.1**	**21.04**	***<0.001***	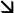
		**O**	**11.5**	**0.0**	**−11.5**	**83.00**	***<0.001***	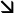
		**G**	**13.3**	**24.6**	**11.3**	**24.03**	***<0.001***	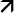
Hand-reared starlings	exp.	**C'**	**0.0**	**8.1**	**8.1**	**43.50**	***<0,001***	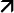
		**E'**	**0.0**	**17.1**	**17.1**	**113.78**	***<0,001***	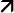
		**B'**	**0.1**	**2.8**	**2.6**	**17.19**	***<0,001***	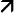
		**A'**	**1.7**	**3.3**	**1.7**	**4.00**	***<0,05***	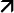
		**D'**	**8.0**	**21.4**	**13.4**	**35.01**	***<0,001***	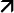
	control	**G'**	**1.8**	**18.2**	**16.4**	**86.09**	***<0,001***	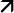
		**H'**	**2.1**	**41.5**	**39.4**	**256.87**	***<0,001***	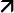
		I'	10.2	9.3	−0.9	0.27	*>0,05*	=
		F'	10.5	7.3	−3.2	3.76	*>0,05*	=

Chi-square tests were made on the absolute values.

#### (ii) Hand-reared starlings

Hand-reared starlings' mean rates of SBs and ARBs were significantly lower in the experimental group than in the control group on the first day (respectively *M*±*SE* = 1.95±1.53% and *M*±*SE* = 6.12±2.43%, Mann-Whitney U test, *p* = 0.049) but not on the last day of the experiment (respectively *M*±*SE* = 10.53±3.73% and *M*±*SE* = 19.09±7.84%, Mann-Whitney U test, *p* = 0.33). This was related to the fact that SBs and ARBs rates increased significantly between the first and the last day of the experiment for the experimental group (Wilcoxon signed rank test, *p* = 0.04) but not for the control group (Wilcoxon signed rank test, *p* = 0.46) (see Figure 2 and Table 1).

#### (iii) Comparison between wild-caught and hand-reared starlings

On the first day of the experiment, the mean SBs and ARBs rates of the experimental hand-reared starlings was significantly lower than that of the experimental wild-caught starlings (Mann-Whitney U test, *p* = 0.02). However, this difference was no longer observed at the end of the experiment (Mann-Whitney U test, *p* = 0.76) (Figure 2). This reflected opposite changes in SBs and ARBs rates in these two experimental groups: whereas SBs and ARBs rates significantly decreased for 7 of the 10 wild-caught starlings, they significantly increased for all the hand-reared starlings (Fisher's exact test comparing the proportions of starlings showing a significant decrease in SBs and ARBs rates in both groups, p = 0.026) (Table 1).

No significant difference was evidenced between control hand-reared starlings and control wild-caught starlings (Mann-Whitney U test, *p*>0.99 on day 1 and p = 0.77 on day 5) (Figure 2).

### 2) Qualitative analysis

**Figure 4:**
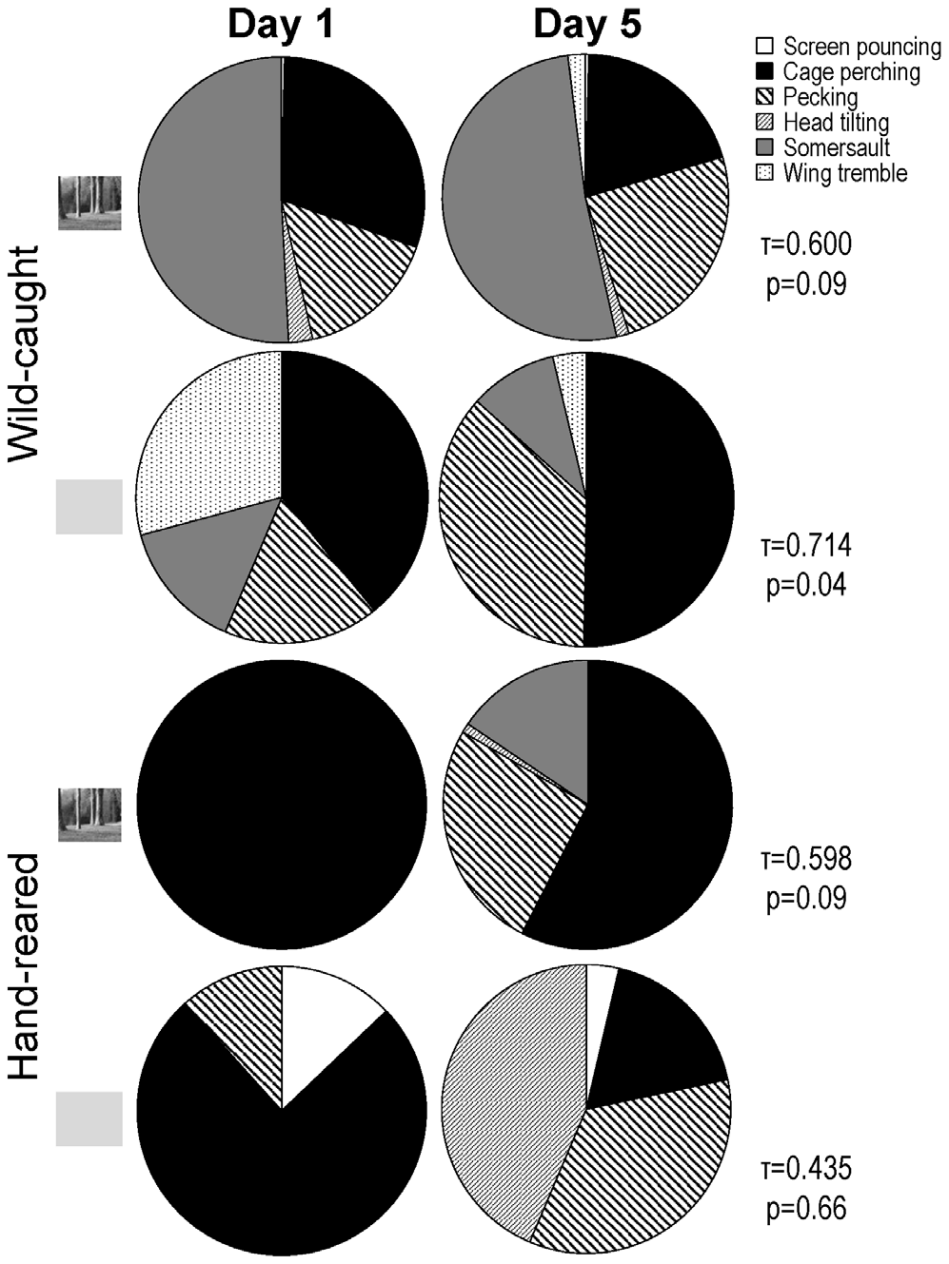
Relative rate (mean percentage) of each type of SBs and ARBs on the first and on the last day of the experiment for experimental and control wild-caught and hand-reared starlings. Small inserts on the left indicate whether the birds were presented landscape videos or grey screen. Values on the right indicate Kendall rank correlation coefficients (τ) and their associated p values.

The types of SBs and ARBs exhibited by the different groups of starlings changed between the first day and the last day of the experiment (Figure 4). Nevertheless, the most frequent SBs and ARBs differed according to treatment. Whereas somersaults were constantly the most frequent SB in experimental wild-caught starlings, the most frequent ARB of control wild-caught starlings was repetitive cage perching. For hand-reared starlings, repetitive cage perching was by far the most frequent (if not the only) ARB in the experimental group. On the first day of the experiment, this was also the most frequent ARB in the control group but, on the last day of the experiment, repetitive pecking and head tilting were the most frequent ARBs in this group. Interestingly, whereas somersaults were never observed in control hand-reared starlings, all but one experimental hand-reared starling exhibited somersaults on the last day of the experiment (although none did on the first day of the experiment).

## Discussion

The present study evaluated the effects of video presentations of natural landscapes on European starlings' SBs and other ARBs. We expected the videos to create an illusion of space, thus reducing the escape motivation of starlings, and consequently decreasing SBs and ARBs rates. Our results only partly support this hypothesis. Although SBs and ARBs rates decreased significantly in most of the experimental wild-caught starlings, this reduction appeared to be restricted to individuals that exhibited high rates of SBs and ARBs on the first day of exposure to videos of landscapes. This suggests that video presentations of natural landscapes are likely to modulate high rates of SBs and ARBs rather than prevent the emergence and development of SBs and ARBs in impoverished conditions.

The fact that SBs and ARBs exhibited by our subjects seem to have shown up spontaneously on the first day or developed in a span of only five days might be surprising. However, it has been shown that starlings can exhibit stereotypic behaviours within a week of being placed in a cage [20]. Moreover, since our wild-caught starlings had been captive for more than 4 years before the experiment began, one can imagine that they had already expressed SBs or ARBs in other contexts, especially those who experienced temporary housing in individual cages. However, when the experiment began, they were group housed in large aviaries, a context in which earlier studies did not detect SBs or ARBSs (e.g. [32], [33]).

One can wonder whether starlings could really perceive the video landscapes. Although we do not know how starlings actually perceive videos, there has been evidence that video presentations can mimic the presence of conspecifics in a variety of avian species, including passerines [26]–[29]. Moreover, since starlings are able to discriminate pictures of landscapes from pictures of conspecifics (unpublished data) and pictures of familiar conspecifics from pictures of unfamiliar conspecifics [30], we know that TFT screens are not a problem for them. It is therefore unlikely that our starlings could simply not perceive the video landscapes. One explanation to the limited effects we observed could be that wild-caught starlings actually perceived the landscapes but somehow neglected the information, maybe because they were too stressed by being maintained singly in a secluded and confined space. Somewhat similar results were obtained in non-human primates whose initial interest for videos rapidly vanished and whose stereotypic behaviours rarely steadily decreased (e.g. [8], [9], [34]).

This study also evaluated the impact of experience on starlings' reactions. According to previous findings [23], [24], we expected hand-reared starlings to perform less SBs and ARBs than wild-caught starlings at the beginning of the experiment, when exposed to a novel situation. However, as they had never been outdoors, we predicted no long-term effect of the videos. In agreement with these expectations, the experimental hand-reared starlings' rate of SBs and ARBs was significantly lower than that of experimental wild-caught starlings on the first day of the experiment. This could be due to experience difference but also to age difference as wild-caught starlings were older than hand-reared starlings. After five days of exposure to videos or to a grey screen, the SBs and ARBs rates of both groups of hand-reared starlings increased. Video presentations thus did not seem to act as a powerful environmental enrichment. Although they appeared to have some positive effect (i.e., the mean SBs and ARBs rate of the experimental group was significantly lower than that of the control group on the first day of the experiment), they were inefficient to prevent permanently the emergence and the development of SBs and other ARBs, as isolation and the social and spatial restrictions may have become more and more frustrating over time. However, it will be necessary to study larger groups of subjects to confirm this idea.

Overall, video presentations of landscapes thus do not seem to be a very powerful environmental enrichment for captive starlings. A qualitative analysis of the different types of SBs and ARBs revealed that these videos may even promote somersaulting. Somersaults were the most frequent SBs exhibited by experimental wild-caught starlings, and, whereas no hand-reared subject performed somersaults on the first day of the experiment, all but one experimental hand-reared starling did on the last day of the experiment. This last result is quite striking, as somersaulting by hand-reared starlings had never been reported previously (e.g. [20]).

How can these variations of the expression of stereotypic behaviours be explained in relation to visual stimuli? According to Feenders and Bateson [20], both somersaulting and repetitive cage perching are strongly linked to escape motivation. However, our results suggest that these two behaviours express different degrees of this motivation; particularly that somersaulting expresses a greater tendency to attempt to escape than repetitive cage perching. Cage perching by itself is not considered stereotypic; only the frenetic repetition of this activity makes it an abnormal behaviour. By contrast, somersaulting is a complex behaviour clearly defined as stereotypic as soon as it is performed by captive birds. In the current study, we found that experimental wild-caught starlings performed more somersaults than control wild-caught starlings did. If somersaults indeed develop from thwarted escape attempts as suggested by Feenders and Bateson [20], one could therefore imagine that, by creating an illusion of outside window or at least providing the only route of escape in an otherwise solid-sided environment, the projection of video landscapes induced a greater escape motivation and thus promoted rather than prevented some types of stereotypies, by increasing the starlings' frustration linked to captivity. Besides, the fact that we observed somersaulting by hand-reared starlings that were exposed to the video landscapes suggests that these particular visual stimulations could also elicit escape motivation in birds that never experienced outdoor life. However, since we do not know what starlings actually perceived in the videos, it could also be that they were just trying to avoid video presentations that appeared to them as odd. Whatever happened, video presentations of landscapes did have an effect on starlings' behaviour, independently of their experience.

## Conclusions

This study reveals that, even if hypothetically realistic, video presentations of natural landscapes have limited positive effects on European starlings' (*Sturnus vulgaris*) SBs and other ARBs. They seem to be more efficient as a remedy to high rates of SBs and ARBs than as an enrichment (and thus as a way to prevent the emergence and development of SBs and ARBs in impoverished conditions). They can even promote some types of SBs putatively linked to escape motivation, independently of outdoor experience. These results highlight the importance of investigating SBs and ARBs not only in quantitative but also in qualitative terms, as it can provide crucial clues on animal welfare.
